# Supplementary material for: The neutrotime transcriptional signature defines a single continuum of neutrophils across biological compartments
Source: Nat Commun. 2021 May 17;12:2856. doi: 10.1038/s41467-021-22973-9 (PMC8129206; doi:10.1038/s41467-021-22973-9)
Supplement: Supplementary file 1 — Supplementary Information [file 41467_2021_22973_MOESM1_ESM.pdf]

The neutrotime transcriptional signature defines a single continuum of neutrophils across biological compartments

Ricardo Grieshaber-Bouyer[1,2], Felix A. Radtke[1,2], Pierre Cunin[1], Giuseppina Stifano[1], Anaïs Levescot[1], Brinda Vijaykumar[3], Nathan Nelson-Maney[1], Rachel B. Blaustein[1], Paul A. Monach[1,4], **Peter A. Nigrovic**[1,5]* and The Immunological Genome Project Consortium

1 Division of Rheumatology, Inflammation, and Immunity, Brigham and Women's Hospital, Harvard Medical School, Boston MA, USA

2 Department of Medicine V, Hematology, Oncology and Rheumatology, Heidelberg University Hospital, Heidelberg, Germany

3 Division of Immunology, Department of Microbiology and Immunobiology, Harvard Medical School, Boston MA, USA

4 Rheumatology Section, VA Boston Healthcare System, Boston MA, USA

5 Division of Immunology, Boston Children's Hospital, Boston MA, USA

These authors jointly supervised this work: Paul A. Monach, Peter A. Nigrovic

* Corresponding Author: Peter A. Nigrovic, MD
Division of Immunology. Boston Children's Hospital
Karp Family Research Building, 10th Floor
1 Blackfan Circle
Boston, MA 02115
Ph: office 617-525-1031, cell 617-905-1373
Fax: 617-525-1010
Email: peter.nigrovic@childrens.harvard.edu

Panel	Channel	Marker	Clone	Vendor	Catalog #	Dilution
Cell sorting	PI	Propidium Iodide	N/A	Sigma-Aldrich	P4170	5 ng/ml
Cell sorting	BV421	CD11b	M1/70	BioLegend	101251	1:100
Cell sorting	AF647	Ly6G	1A8	BioLegend	127610	1:100
IL-1β inflammation	AF350	LIVE/DEAD	N/A	ThermoFisher	L23105	According to instructions
IL1β inflammation	BV421	CD9	KMC8	BD	564235	1:100
IL1β inflammation	BV510	CXCR4	2B11/CXCR4	BD	563468	1:100
IL1β inflammation	BV605	CD11b	M1/70	BioLegend	101237	1:100
IL1β inflammation	FITC	CD53	OX-79	BioLegend	124705	1:100
IL1β inflammation	PerCP-eFluor710	CD14	Sa14-2	BioLegend	123313	1:100
IL1β inflammation	PE	CD33	9A11-CD33	ThermoFisher	12-0331-82	1:100
IL1β inflammation	APC	CCR1	S15040E	BioLegend	152503	1:100
IL1β inflammation	AF700	Ly6G	1A8	BioLegend	127621	1:100
IL1β inflammation	APC-Cy7	CXCR2	SA044G4	BioLegend	149313	1:100
IL1β inflammation	BV421	Rat IgG2a, κ	R35-95	BD	562602	1:100
IL1β inflammation	BV510	Rat IgG2b, κ	R35-38	BD	562951	1:100
IL1β inflammation	BV605	Rat IgG2b, κ	RTK4530	BioLegend	400649	1:100
IL1β inflammation	FITC	Rat IgM, κ	RTK2118	BioLegend	400805	1:100
IL1β inflammation	PerCP-eFluor710	Rat IgG2a, κ	RTK2758	BioLegend	400531	1:100
IL1β inflammation	PE	Rat IgG1, κ	eBRG1	ThermoFisher	12-4301-82	1:100
IL1β inflammation	APC	Rat IgG2b, κ	RTK4530	BioLegend	400611	1:100
IL1β inflammation	AF700	Rat IgG2a, κ	RTK2758	BioLegend	400528	1:100
IL1β inflammation	APC-Cy7	Rat IgG2a, κ	RTK2758	BioLegend	400523	1:100
K/BxN model	AF350	LIVE/DEAD	N/A	ThermoFisher	L23105	According to instructions
K/BxN model	BV421	CD9	KMC8	BD	564235	1:100
K/BxN model	BV510	CXCR4	2B11/CXCR4	BD	563468	1:100
K/BxN model	BV605	CD11b	M1/70	BioLegend	101237	1:100
K/BxN model	FITC	CD53	OX-79	BioLegend	124705	1:100
K/BxN model	PerCP-eFluor710	CD14	Sa14-2	BioLegend	123313	1:100
K/BxN model	PE	CD63	NVG-2	BioLegend	143903	1:100
K/BxN model	AF647	Ly6G	1A8	BioLegend	127609	1:100
K/BxN model	APC-Cy7	CXCR2	SA044G4	BioLegend	149313	1:100
K/BxN model	BV421	Rat IgG2a, κ	R35-95	BD	562602	1:100
K/BxN model	BV510	Rat IgG2b, κ	R35-38	BD	562951	1:100
K/BxN model	BV605	Rat IgG2b, κ	RTK4530	BioLegend	400649	1:100
K/BxN model	FITC	Rat IgM, κ	RTK2118	BioLegend	400805	1:100
K/BxN model	PerCP-eFluor710	Rat IgG2a, κ	RTK2758	BioLegend	400531	1:100
K/BxN model	PE	Rat IgG2a, κ	RTK2758	BioLegend	400507	1:100
K/BxN model	AF647	Rat IgG2a, κ	RTK2758	BioLegend	400526	1:100
K/BxN model	APC-Cy7	Rat IgG2a, κ	RTK2758	BioLegend	400523	1:100

Supplementary Table 1. Flow cytometry panels used for cell sorting and confirmation studies.

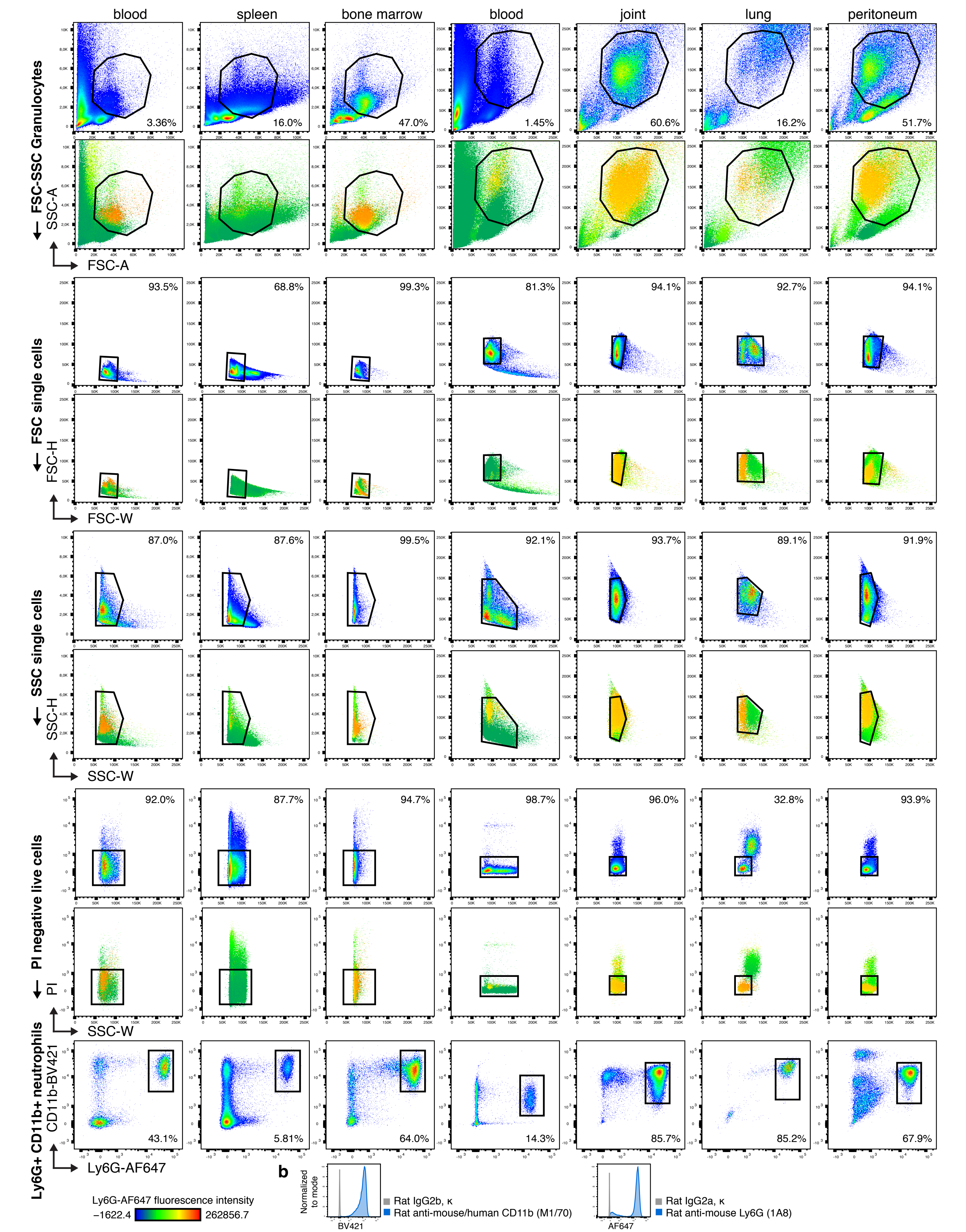

Supplementary Figure 1 | Detailed overview of the flow cytometry gating and sorting strategy in all tissues.
a, Flow cytometry gating strategy used in analysis and to sort live neutrophils. Cells were gated as FSC/SSC granulocytes, FSC and SSC single cells, PI negative cells and Ly6G-positive CD11b-positive neutrophils. Coloring of cells alternates between density and expression of Ly6G-AF647. For sorting, cells from N = 3 mice were pooled for each tissue in each experiment. **b**, Isotype controls were used to determine sort cutoffs.

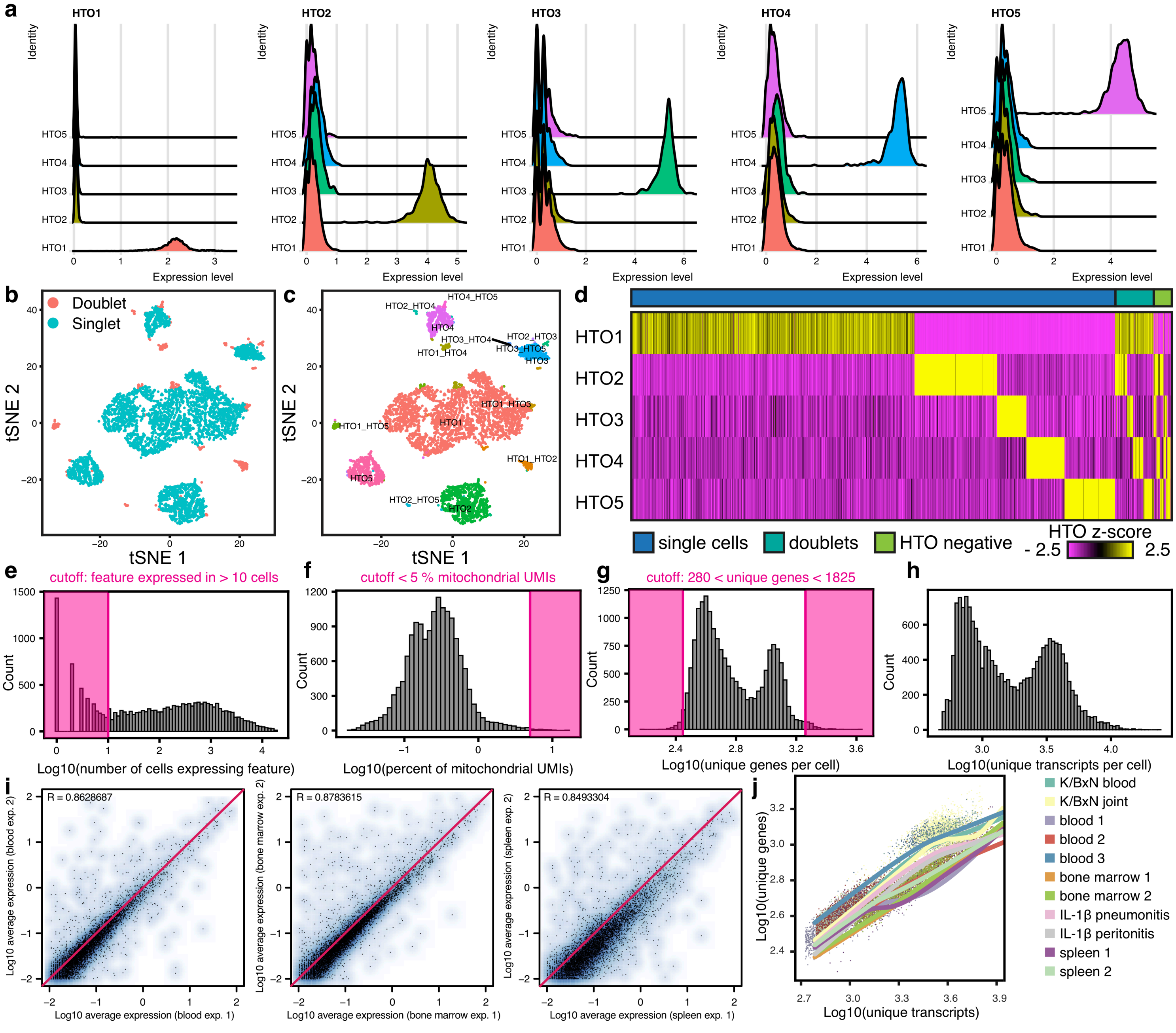

Supplementary Figure 2 | Detailed overview of the deconvolution of Hashtag oligonucleotide (HTO)-based multiplexing and scRNA-seq quality control.

a, Representation of all HTO expression signals for each confidently assigned HTO population shows minimal signal in all channels except the assigned channel. **b**, tSNE embedding of HTO expression colored by doublet status shows cell doublets at the edges of stable HTO clusters. **c**, tSNE embedding of HTO expression colored by HTO assignment shows pure HTO 1–5 clusters and mixed clusters, which were excluded from downstream analysis. **d**, Heatmap of HTO signals for single cells showing clean HTO 1–5 populations, cell doublets and HTO-negative events (below automatically chosen threshold). **e**, Distribution of total gene expression for each gene in all cells. **f**, Distribution of the fraction of mitochondrial transcripts in all counted transcripts. **g**, Distribution of the number of unique genes per cell. **h**, Distribution of unique molecular identifiers per cell. **i**, Convergence between datasets enabling a combined analysis by aligning the datasets. **j**, Relationship between the number of detected transcripts and the number of detected genes per cell.

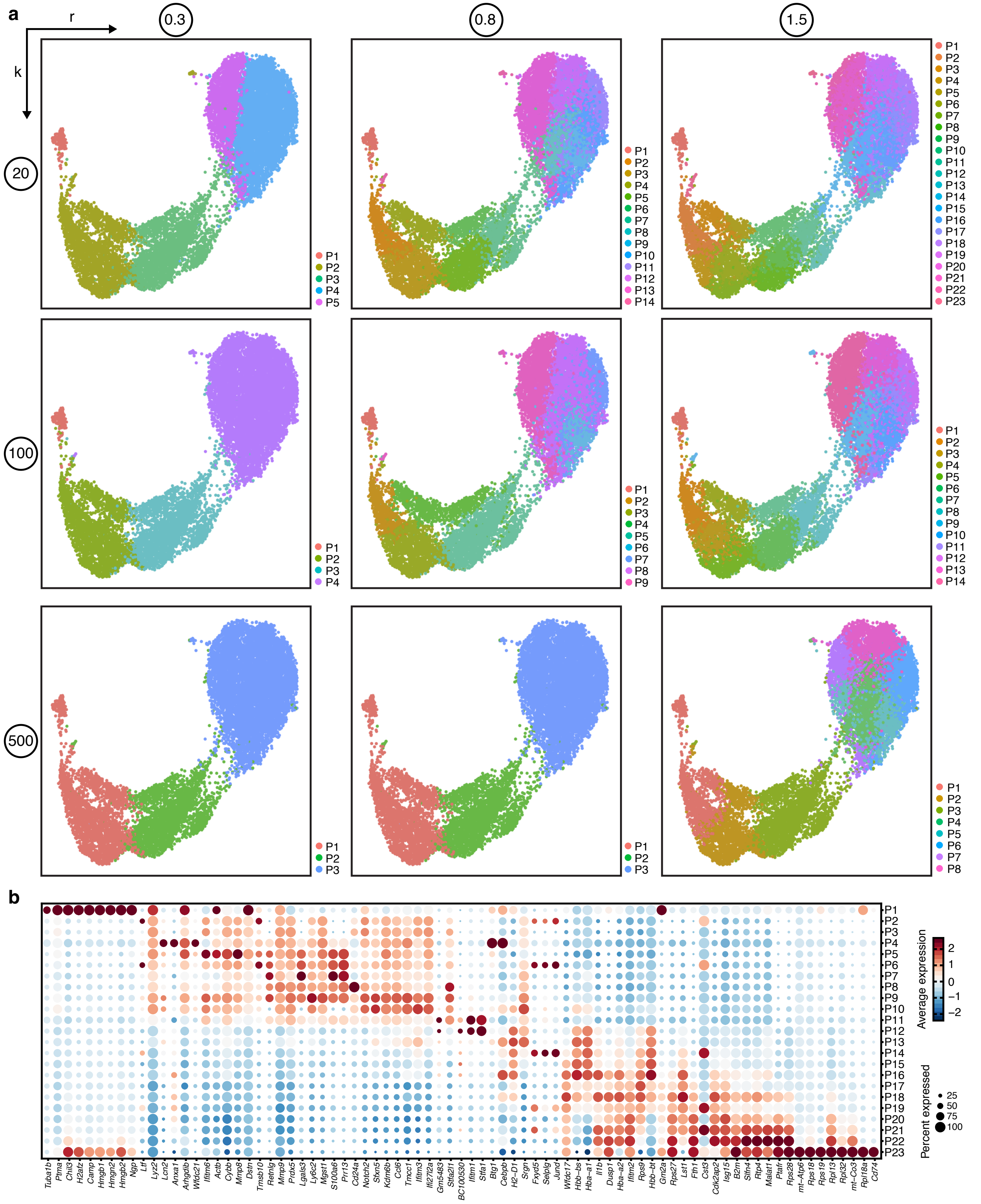

Supplementary Figure 3 l Instability of conventional cluster-based dimensionality reduction techniques.
a, UMAP plots followed by community detection varying the necessary a priori assumptions of k nearest neighbors and the coarseness of resolution within reasonable boundaries (k = 20 – 500; r = 0.3 – 1.5). **b**, Visualization of marker genes for the detected clusters of neutrophils suggest a gradual transcriptomic shift instead of sharp distinctions between populations.

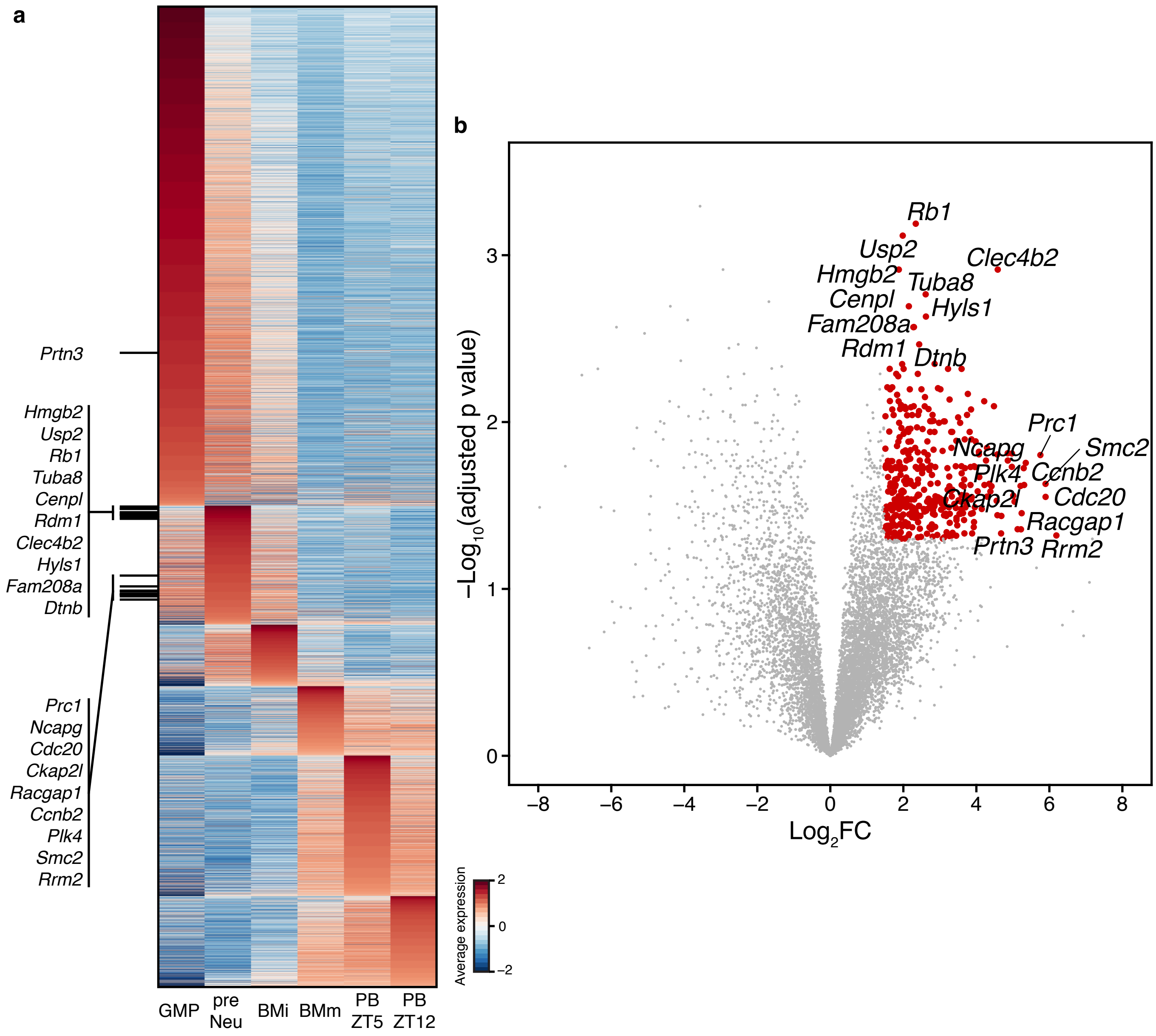

Supplementary Figure 4 I Gene expression signature of preNeus.
a, Heatmap visualizing z-scaled gene expression in developmental stages of neutrophils. **b**, Volcano plot showing differentially expressed genes between preNeus and all other neutrophil samples. Limma was used to model each gene as a linear model with samples assigned as either preNeu or all other groups. P-values were Benjamini-Hochberg corrected (corresponding to the false discovery rate); cutoff: 0.05 and an absolute $\log_2$-fold-change ≥ 1.5. Genes selected for calculation of the preNeu score are highlighted in red. GMP = Granulocyte monocyte precursor, preNeu = committed proliferative neutrophil precursor, BMi = immature bone marrow neutrophil, BMm = mature bone marrow neutrophil, PB ZT5 = peripheral blood neutrophil zeitgeber time 5 hours, PB ZT12 = peripheral blood neutrophil zeitgeber time 12 hours.

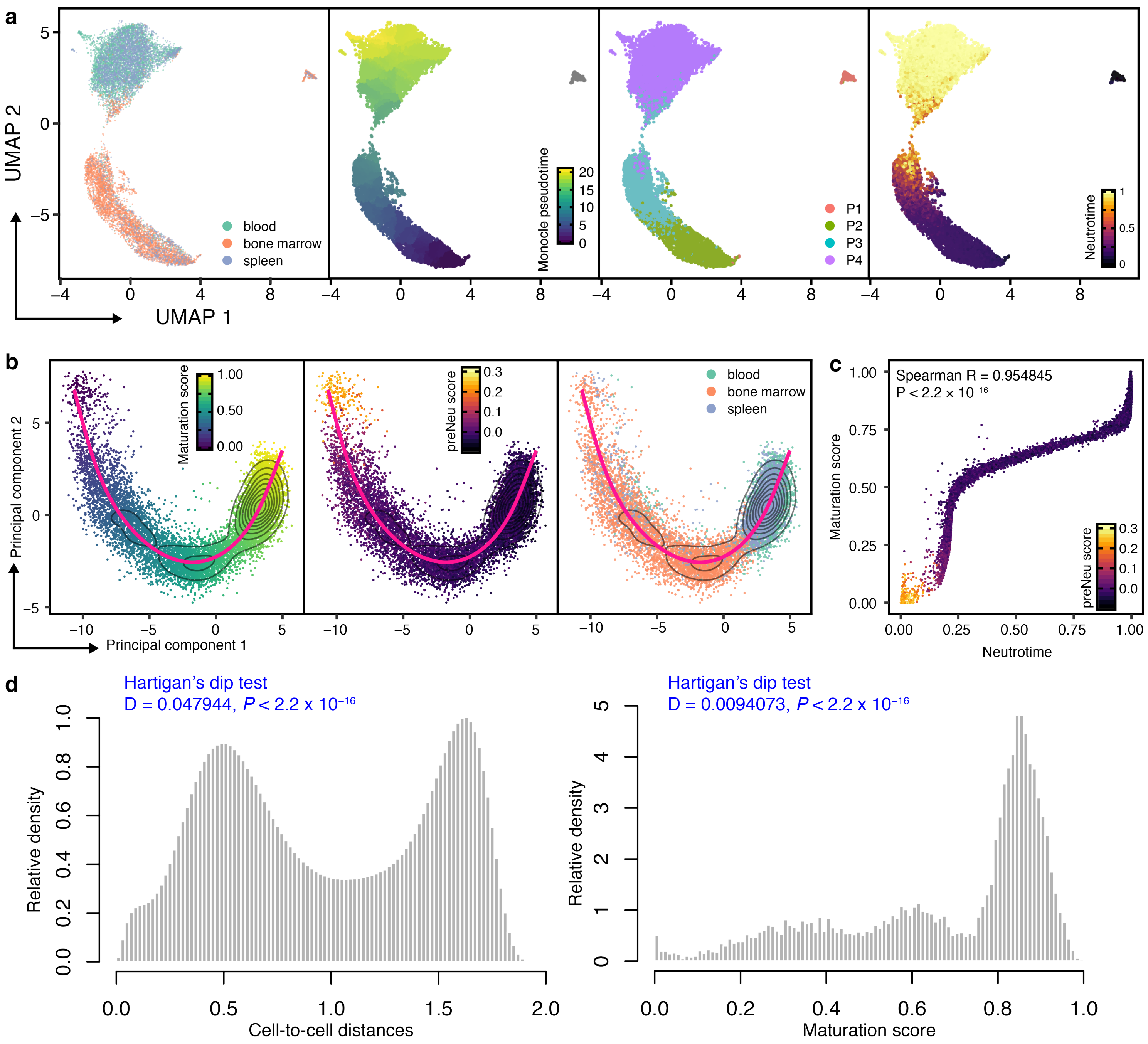

Supplementary Figure 5 I Different dimensionality reduction techniques achieve similar results to neutrotime.
a, Dimensionality reduction in Monocle 3 and trajectory analysis. **b**, Principal component embedding of all healthy neutrophils with a principal curve; contour plots reflect numeric abundance. **c**, Concordance between the principal curve and neutrotime. **d**, Hartigan's dip test applied to a cell-to-cell distance matrix (calculated from the first 20 principal components as 1 – Pearson correlation) and to the maturation score from the principal curve.

a GO terms enriched in early neutrotime

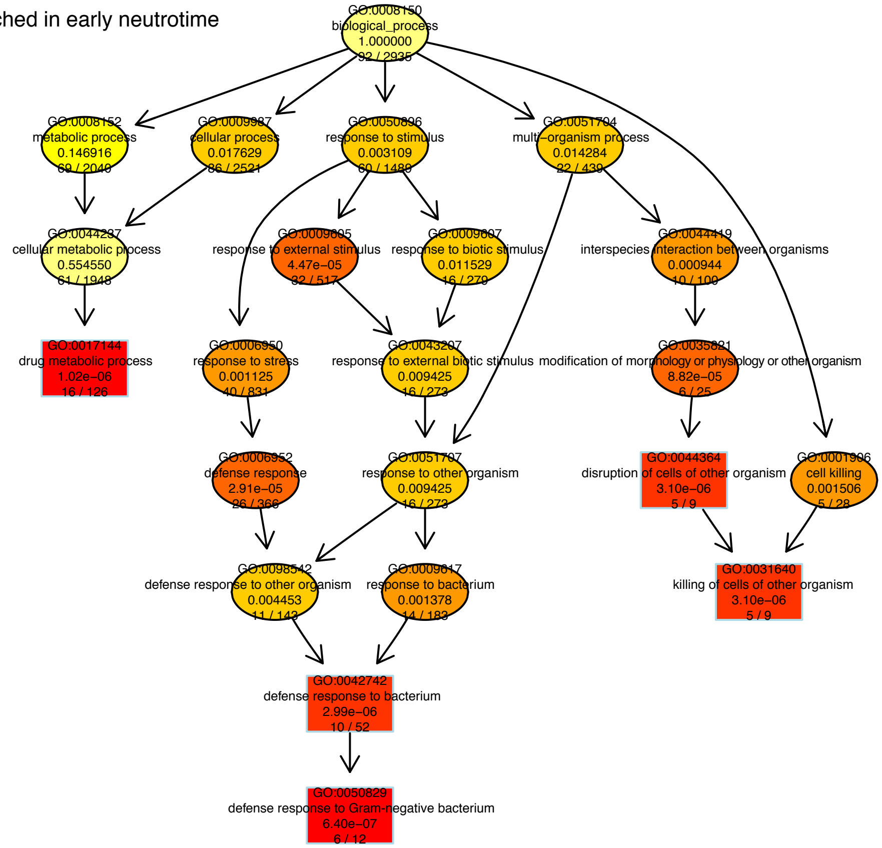

b GO terms enriched in late neutrotime

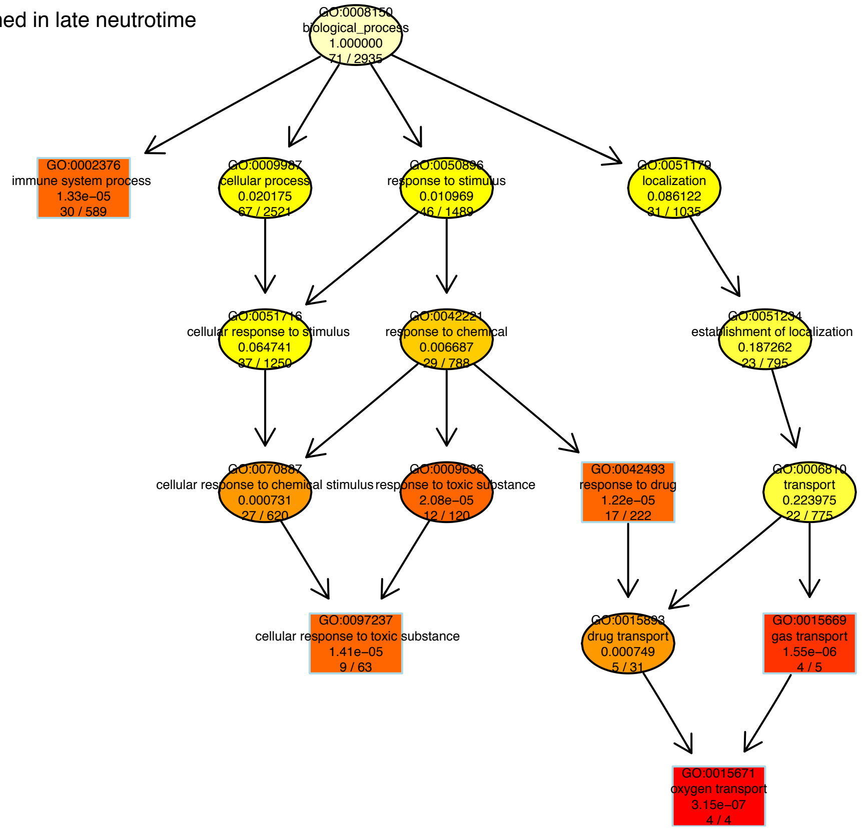

Supplementary Figure 6 | Gene Ontology (GO) term analysis of transcriptomes along neutrotime.
a, Highly enriched GO terms in early neutrotime. **b**, Highly enriched GO terms in late neutrotime.

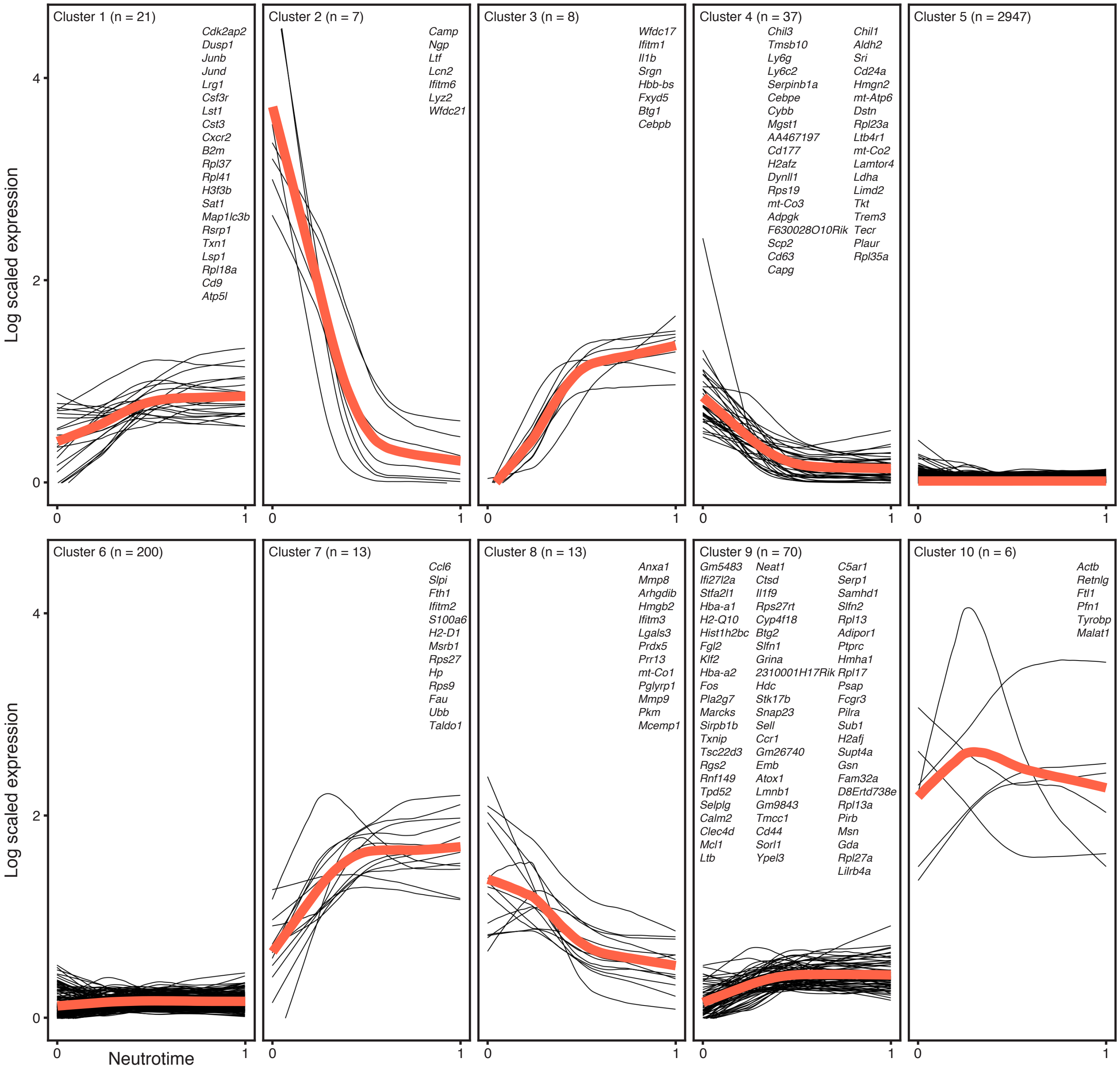

Supplementary Figure 7 | k-means clustering of gene expression patterns along neutrotime.
Red line indicates the centroid of each cluster. Cells are ordered along neutrotime (same scale as **Fig. 2g**).

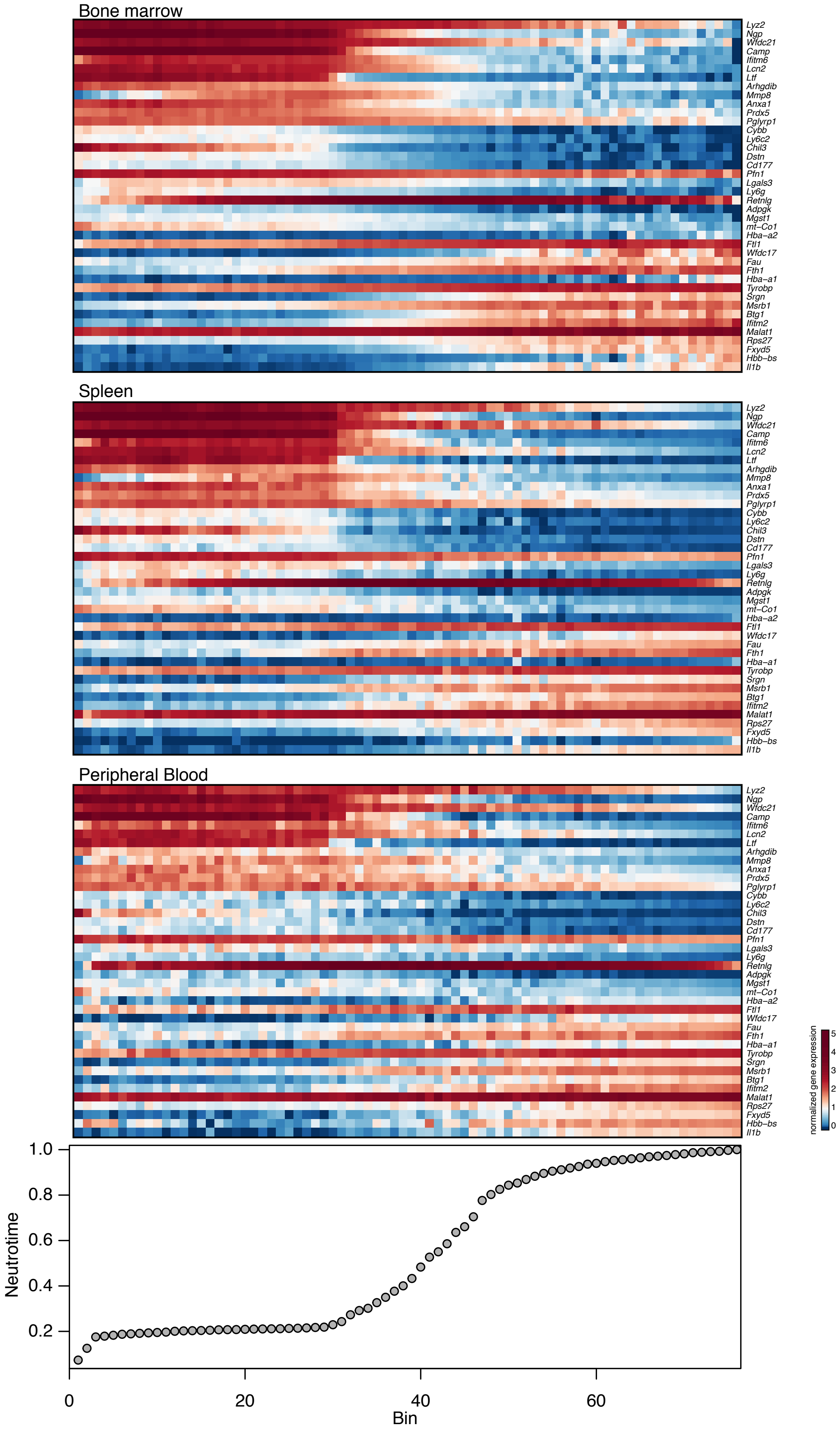

Supplementary Figure 8 I Neutrotime gene expression across different tissues.
Heatmap representation of relative expression of core neutrotime genes split by each tissue. Cells were ordered along neutrotime and binned using a continuous increase in neutrotime (0.00001) smaller than the smallest difference of neutrotime between two cells until at least 5 cells per tissue and at least 30 cells total were represented in each respective bin. This approach yielded a continuous collection of cells in 76 bins. Lower panel shows the neutrotime stage for each bin.

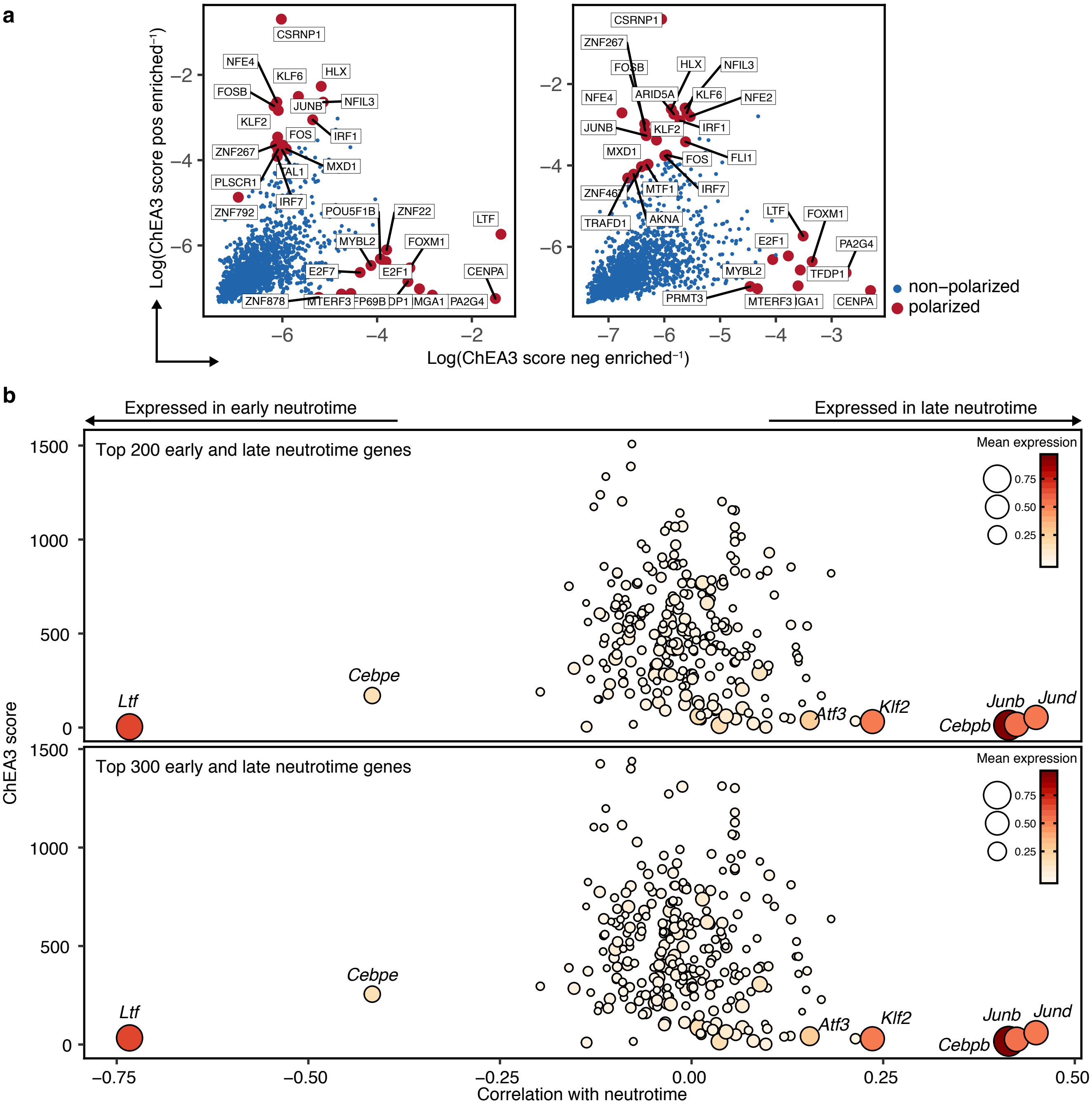

Supplementary Figure 9 I Combined ChEA3 and TF expression analysis with varying numbers of genes.
a, Inferred transcriptional regulators of early and late neutrotime via ChEA3 using the top 200 (left) or top 300 (right) early and late neutrotime genes as basis to calculate ChEA3 scores. Higher $\log_e$(ChEA3 score neg enriched^{-1}) indicates greater activity in early neutrotime, while higher $\log_e$(ChEA3 score pos enriched^{-1}) indicates greater activity in late neutrotime. Since ChEA3 combines murine, human and rat libraries, regulators were shown with the human protein symbols. **b**, Inferred regulatory activity versus transcription factor expression along neutrotime using the top 200 or top 300 early and late neutrotime genes.

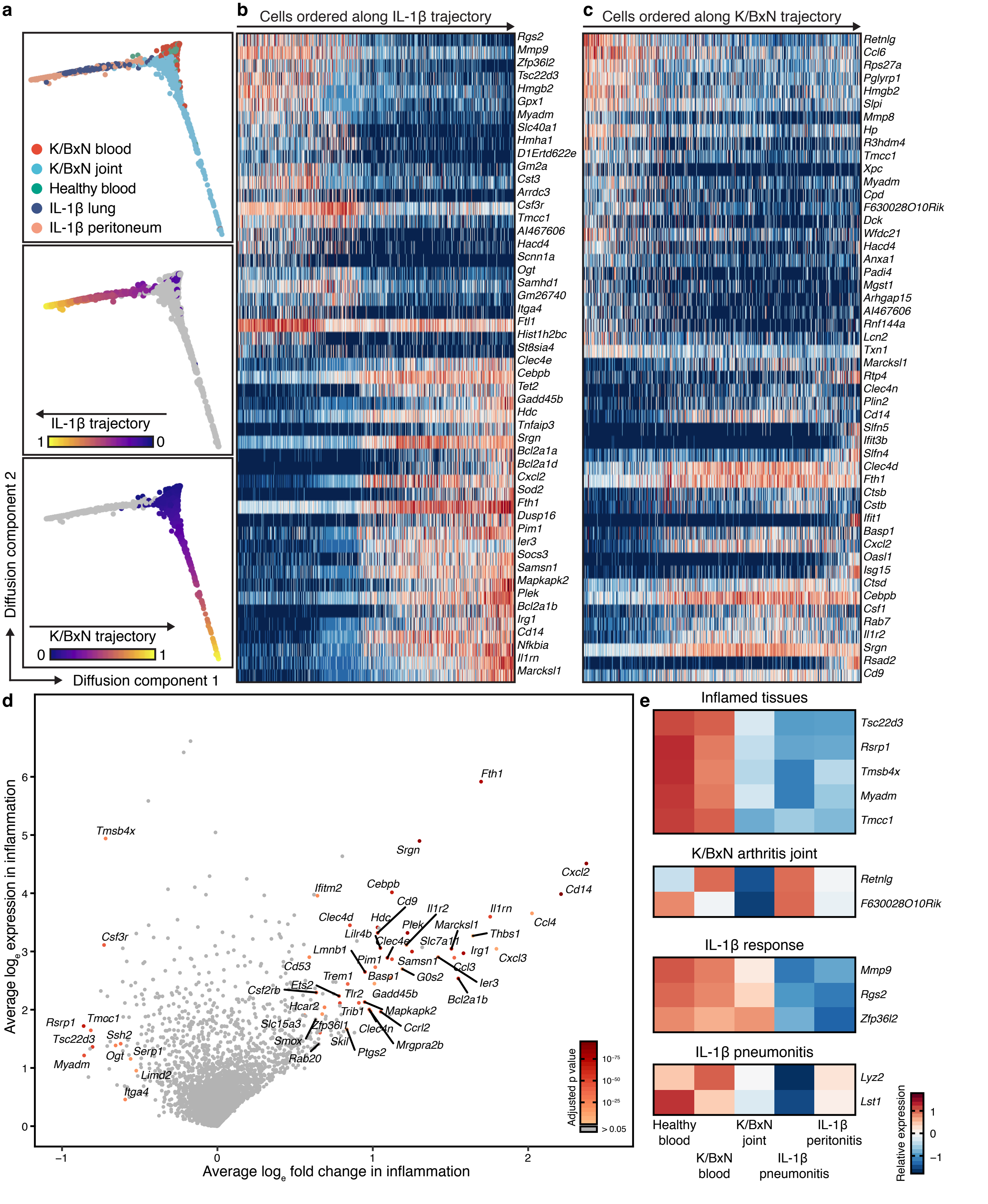

Supplementary Figure 10 | Neutrophils in inflammation.
a, Diffusion map embedding of neutrophils from inflamed compartments highlights a divergence between acute IL-1β-induced (pneumonitis and peritonitis) and subacute (K/BxN serum transfer arthritis) inflammation. **b**, Gene expression changes in neutrophils ordered along the IL-1β and (**c**) K/BxN trajectory. **d**, Identification of a canonical inflammatory program in neutrophils from inflamed joint, lung and peritoneum compared to healthy blood. The least significant of the adjusted P values of the three pairwise comparisons was chosen as conservative approach. Shown is the average $\log_e$ normalized expression and average $\log_e$ fold change in inflammation compared to healthy peripheral blood. **e**, Heatmap of downregulated genes in inflammation.

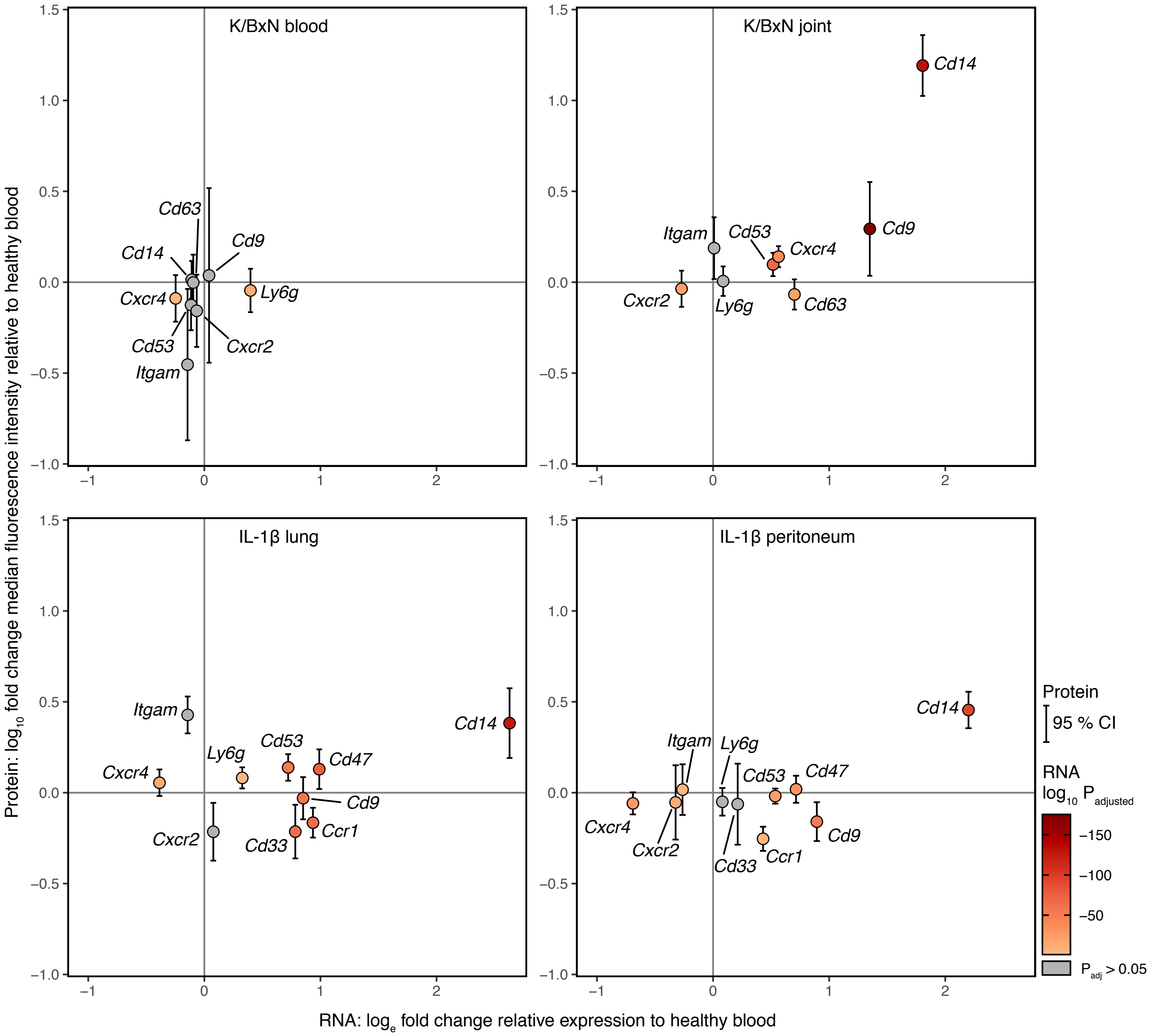

Supplementary Figure 11 I Gene and protein expression changes in inflammation.
Neutrophils in healthy tissues and models of experimental inflammation were stained with antibodies directed against proteins differentially expressed between healthy and inflamed neutrophils (Fig. 7). Protein expression represent the average of 5 mice (4 in peritonitis) per condition. The $\log_e$ fold change compared to healthy peripheral blood for RNA and $\log_{10}$ protein expression (mean fluorescence intensity) is shown. Vertical error bars indicate the 95 % confidence interval for protein change.
