# Supplementary material for: The neutrotime transcriptional signature defines a single continuum of neutrophils across biological compartments
Source: Nat Commun. 2021 May 17;12:2856. doi: 10.1038/s41467-021-22973-9 (PMC8129206; doi:10.1038/s41467-021-22973-9)
Supplement: Supplementary file 3 — Description of Additional Supplementary Files [file 41467_2021_22973_MOESM3_ESM.pdf]

Description of Additional Supplementary Files

File Name: Supplementary Data 1
Description: A table containing differentially expressed genes in murine neutrophils across the tested inflammatory conditions.

File Name: Supplementary Data 2
Description: A table containing microarray gene expression data for 249 ImmGen cell populations used to assign single cells to reference populations. Data obtained from www.immgen.org.

File Name: Supplementary Data 3
Description: A table containing gene expression changes (fold change) in immune lineages 2 hours after subcutaneous injection of 10,000 IU IFNα in mice.
Data obtained from reference 35: Mostafavi, S. et al. Parsing the Interferon Transcriptional Network and Its Disease Associations. *Cell* 164, 564–578 (2016).

File Name: Supplementary Data 4
Description: Table containing murine and human ortholog genes and Spearman correlation values for each gene with the neutrotime signature.
